# Tracheal Glomus Tumor: A Case Report with CT Imaging Features

**DOI:** 10.3390/medicina58060791

**Published:** 2022-06-13

**Authors:** Jeong-A Yeom, Yeon-Joo Jeong, Hyo-Yeong Ahn, Jung-Seop Eom, Chang-Hun Lee, Min-Hee Hwang

**Affiliations:** 1 Department of Radiology, Pusan National University Yangsan Hospital, Yangsan-si 50612, Korea; mdyja@naver.com; 2 Department of Radiology, Biomedical Research Institute, Pusan National University Hospital, Pusan National University School of Medicine, Busan 49241, Korea; lunar9052@hanmail.net; 3 Department of Thoracic and Cardiovascular Surgery, Medial Research Institute, Pusan National University Hospital, Pusan National University School of Medicine, Busan 49241, Korea; doctorahn02@daum.net; 4 Department of Internal Medicine, Pusan National University School of Medicine, Busan 49241, Korea; ejspulm@gmail.com; 5 Department of Pathology, Medial Research Institute, Pusan National University Hospital, Pusan National University School of Medicine, Busan 49241, Korea; cnlee@pusan.ac.kr

**Keywords:** bronchoscopic biopsy, computed tomography

## Abstract

Background and Objectives: Glomus tumors are rare benign tumors. The majority of them affect the skin the most and are rarer in the trachea, where the glomus bodies may not be present. Only scarce reports of tracheal glomus tumors have been reported solely with case reports of relevant articles. Materials and Methods: A 53-year-old man, with a free previous medial history, presented to our hospital with tracheal mass which was incidentally found. He did not complain of any specific symptoms associated with the tracheal tumor. The contrast-enhanced chest computed tomography (CT) revealed an avid enhancing nodular lesion, which is similar to blood vessels, in the trachea, 3 cm above the carina level without definite airway obstruction. Results: Successful tracheal resection and end-to-end anastomosis were performed on the patients; therefore, the final post-operative pathologic findings revealed a benign tracheal glomus tumor. The follow-up CT scan four months after operation showed complete removal of the tumor. Conclusion: Tracheal glomus tumors, even rare entities, could be considered as a differential diagnosis if a highly enhancing mass appears on CT images.

## 1. Introduction

A glomus tumor is a rare benign tumor that originates from modified smooth muscle cells of normal glomus bodies, which are formed at anastomoses of arteries and veins. They affect blood flow and control body temperature. The tumor can occur anywhere in the body, but for the most part, this tumor affects skin and is rare in the trachea, where normal glomus bodies may not be present. Although clinically, the symptoms range from asymptomatic to severe discomfort, symptoms related to airway irritation are common in bronchial and tracheal glomus tumor [1,2]. Due to its rarity, few data are available on tracheal glomus tumors, and fewer than 80 cases have been reported [2]. In this case report, we focus on its imaging and clinical features and their pathologic corrections. Details of more cases and valid literature reviews are required to enable the diagnostic characteristics of glomus tumors to be accurately identified.

## 2. Case Description

**Figure 1:**
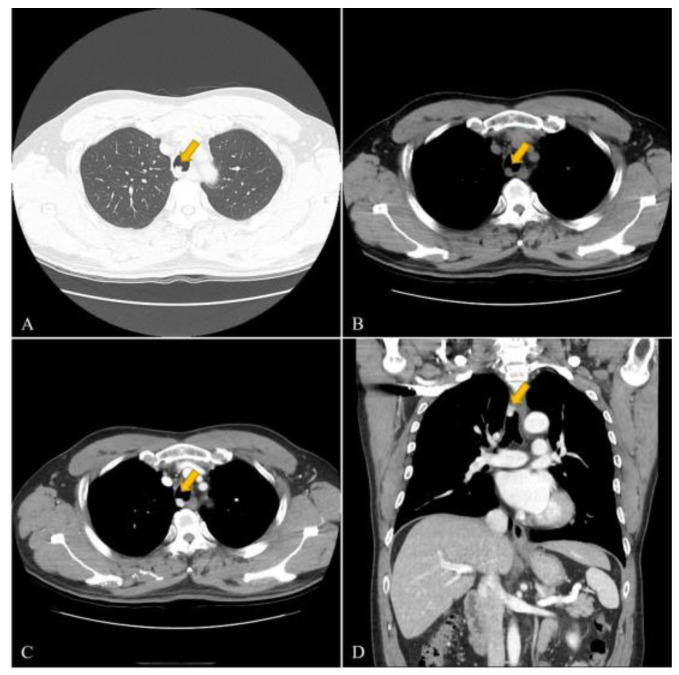
53-year-old male with tracheal glomus tumor. (**A**) CT lung window setting images; (**B**) pre-contrast enhanced image; (**C**) post-contrast enhanced image; and (**D**) coronal post-contrast enhanced image. (**A**) The lung window setting CT image showed a 1.4 × 1.3 cm sized protruding mass in the right posterior wall of the mid trachea; (**B**,**C**) on contrast CT images, the lesion exhibited a high contrast enhancement similar to that of blood vessel; (**D**) the coronal post-contrast-enhanced CT image showed the lesion was located 3 cm above the carina and did not obstruct the airway.

**Figure 2:**
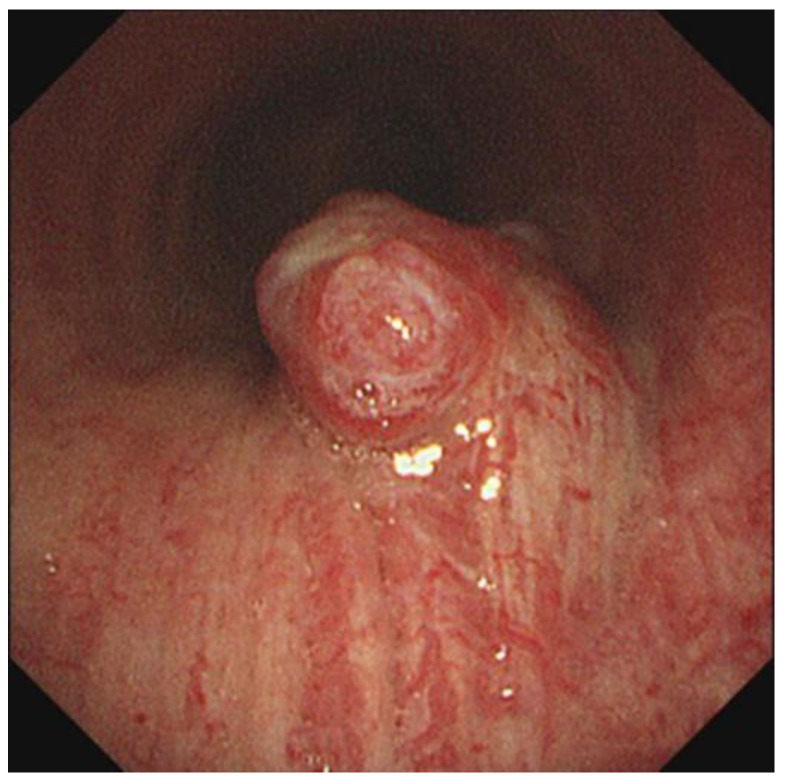
Bronchoscopy revealed a 1.5 cm sized polypoid endotracheal mass with a hyperemic mucosal surface in the mid portion of the trachea.

**Figure 3:**
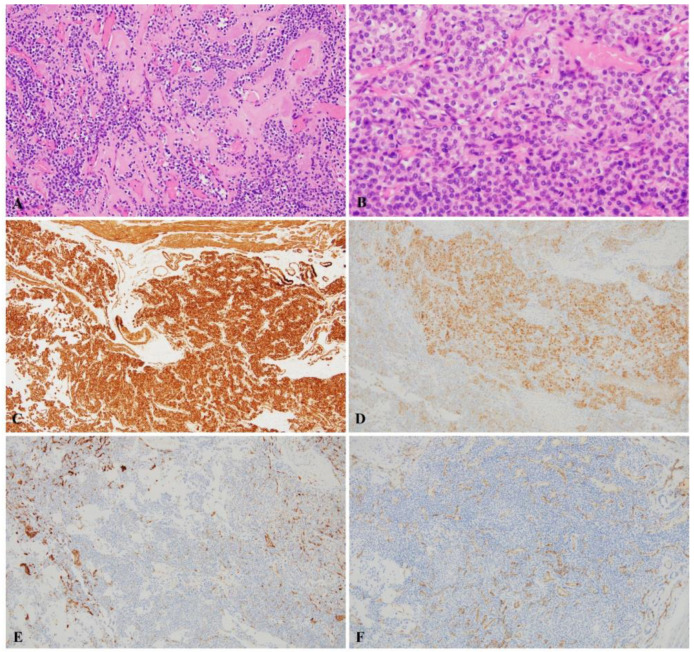
The histopathological findings after surgery showing typical features of tracheal glomus tumor. (**A**) The tumor consisted of a proliferation of epithelioid cells and abundant vascular channels (hematoxylin and eosin [H&E], ×200); (**B**) tumor cells were abundant, single, round, and small-sized, with uniform eosinophilic or transparent cytoplasm and hyperchromatic nuclei and formed microvascular spaces (H&E, ×400); (**C**–**F**) immunohistochemistry showed tumor cells were diffusely positive for smooth muscle actin (SMA), focally positive for synaptophysin (Syn), but negative for CD56 and CD31 (c; SMA, ×100, d; Syn, ×100, e; CD56, ×100, f; CD31 ×100).

A 53-year-old male was referred to our hospital with a diagnosis of a tracheal mass, which was found incidentally during a health-screening check. The patient had no specific clinical symptoms. He was previously healthy and had no relevant medial history. The clinical physical examination revealed no specific findings. His vital signs were stable, with blood pressure 110/70 mmHg, heart rate 78 bpm, respiratory rate 15 breaths per minute, and oxygen saturation in room air 98%. Laboratory findings, including complete blood cell count, liver and thyroid function tests, C-reactive protein (CRP), erythrocyte sedimentation rate (ESR), and urine analysis results were normal. Electrocardiography and pulmonary function test results were also normal. As imaging examinations, chest radiography results at admission were normal (not shown). However, computed tomography (CT) revealed a highly enhancing mass lesion measuring 1.4 × 1.3 × 1.2 cm in the right posterior wall of the mid trachea and 3 cm above carina level without definite evidence of airway obstruction. The mass showed contrast enhancement similar to that of blood vessels (Figure 1). No definite evidence of lymphadenopathy or other pulmonary parenchymal abnormalities was observed. Bronchoscopy was performed to exclude malignancy, and a 1.5 cm sized polypoid endotracheal mass with a hyperemic mucosal surface was observed in the mid portion of the trachea, which was in line with CT findings (Figure 2). Rigid bronchoscopic biopsy was performed under general anesthesia to obtain an accurate diagnosis. However, peri-procedural bleeding required epinephrine to achieve hemostasis before the procedure was completed. According to radiologic and pathologic findings of the bronchoscopic biopsy, the tracheal mass was consistent with glomus tumor of the trachea, and this was confirmed immunohistochemically. After the bronchoscopic biopsy, the patient decided to have the tumor removed in the operating room. The patient was referred to the thoracic and cardiovascular surgery department. Tracheal resection and end to end anastomosis were planned, and the surgery was performed through a right posterolateral thoracotomy after confirmation of the presence of a mass on the posterolateral wall of the trachea, 3 cm above the carina by bronchoscopy in the operating room. There was no definite evidence of an immediate postoperative complication after surgery. The gross pathological findings were a tumor size 2.0 × 1.2 cm and a clear resection margin. Hematoxylin and eosin (H&E) staining showed the tumor was composed of single, round, small cells with uniform, transparent eosinophilic cytoplasm, and hyperchromatic nuclei forming microvascular spaces. Immunohistochemical analysis showed cells were diffusely positive for smooth muscle actin antibody (SMA), focally positive for synaptophysin (Syn), but negative for cytokeratin (CK), chromogranin-A, CD56, and CD31 (Figure 3). The right recurrent laryngeal nerve lymph node specimen obtained during surgery provided no definite evidence of tumor infiltration. The in-hospital postoperative period was uneventful, and the patient was discharged at a time after surgery. Follow-up CT at 4 months postoperatively (not shown) showed the glomus tumor was well removed and there was no definite evidence of other specific abnormal findings.

## 3. Discussion

Glomus tumors are composed of modified perivascular cells that resemble those of normal glomus bodies, which are involved in temperature control by regulating peripheral blood flow [3]. While most glomus tumors present as small masses in soft dermal tissues of the extremities, such as the finger subungual area, they can occur anywhere in the body. The World Health Organization (WHO) classifies glomus tumors into three categories: benign glomus tumors, glomus tumors of uncertain malignant potential, and malignant glomus tumors (Table 1) [4]. Although the majority of reported glomus tumors are benign, a small proportion are histologically malignant [4].

Glomus tumors presenting in airways are extremely rare; the majority are benign [4,5,6] and usually occur in the distal part of the respiratory tree [7,8,9]. The most common symptoms of tracheal glomus tumors include shortness of breath, cough, stridor due to tracheal obstruction, and hemoptysis due to the vascular nidus nature of the tumors. However, glomus tumors are usually asymptomatic [1,3] and are often diagnosed incidentally, as in our patient.

CT provides a useful means of diagnosing glomus tumor arising from the endobronchial tree. On CT images, these tumors are visualized as masses with avid contrast enhancement due to their abundant vascularity, as was observed in our case. When a glomus tumor arises from the respiratory tract, it mimics a variety of disease entities, which range from benign mucosal balls to malignant entities necessitating bronchoscopy [10]. The leading components of the differential diagnosis of tracheal glomus tumor include carcinoid tumor and hemangiopericytoma. All three usually appear as well-circumscribed round masses that are relatively well-enhanced on contrast images. Despite diagnostic difficulties regarding the differentiation of these tumors based on radiologic findings alone, the obvious contrast enhancement on CT images displayed by tracheal glomus tumors is a relatively specific finding, and the histopathologic identification of cytological and vascular structural characteristics aids the differential diagnoses [11,12,13].

Although several adequate methods are suitable for treating tracheal glomus tumors, such as tracheal stenting through bronchoscopy or laser resection with or without adjuvant radiotherapy, surgical resection is the definitive curative treatment of choice and requires no further treatment. The possibility of tumor recurrence after endoscopic removal is higher than that after complete surgical removal, even when tumors appear benign [14].

## 4. Conclusions

Tracheal glomus tumor is an extremely rare entity, but when CT depicts a mass with an avid contrast enhancement pattern in the respiratory tract, this tumor should be considered in the differential diagnosis. Furthermore, CT imaging was found to be an appropriate guide for diagnosis and treatment.

## Figures and Tables

**Table 1:** WHO classification of glomus tumors (5th edition, 2020) ^1^.

Malignant Glomus Tumor	Uncertain Malignant Potential
(1) Marked nuclear atypia and any level of mitotic activity or	Not fulfilling the criteria for malignancy but has one or more atypical features other than nuclear polymorphisms
(2) Having atypical mitotic figures.	e.g., tumor size greater than 2.0 cm and location in a deep site (in the absence of nuclear atypia).

^1^ No significant changes or newly described entities were introduced in the glomus tumor, which includes perivascular, compared with 4th edition, 2013.
